# Resistance to *Bacillus thuringiensis* Mediated by an ABC Transporter Mutation Increases Susceptibility to Toxins from Other Bacteria in an Invasive Insect

**DOI:** 10.1371/journal.ppat.1005450

**Published:** 2016-02-12

**Authors:** Yutao Xiao, Kaiyu Liu, Dandan Zhang, Lingling Gong, Fei He, Mario Soberón, Alejandra Bravo, Bruce E. Tabashnik, Kongming Wu

**Affiliations:** 1 The State Key Laboratory for Biology of Plant Disease and Insect Pests, Institute of Plant Protection, Chinese Academy of Agricultural Sciences, Beijing, China; 2 College of Life Science, Central China Normal University, Wuhan, China; 3 Instituto de Biotecnología, Universidad Nacional Autónoma de México, Cuernavaca, Morelos, Mexico; 4 Department of Entomology, University of Arizona, Tucson, Arizona, United States of America; University College London, UNITED KINGDOM

## Abstract

Evolution of pest resistance reduces the efficacy of insecticidal proteins from the gram-positive bacterium *Bacillus thuringiensis* (Bt) used widely in sprays and transgenic crops. Recent efforts to delay pest adaptation to Bt crops focus primarily on combinations of two or more Bt toxins that kill the same pest, but this approach is often compromised because resistance to one Bt toxin causes cross-resistance to others. Thus, integration of Bt toxins with alternative controls that do not exhibit such cross-resistance is urgently needed. The ideal scenario of negative cross-resistance, where selection for resistance to a Bt toxin increases susceptibility to alternative controls, has been elusive. Here we discovered that selection of the global crop pest, *Helicoverpa armigera*, for >1000-fold resistance to Bt toxin Cry1Ac increased susceptibility to abamectin and spineotram, insecticides derived from the soil bacteria *Streptomyces avermitilis* and *Saccharopolyspora spinosa*, respectively. Resistance to Cry1Ac did not affect susceptibility to the cyclodiene, organophospate, or pyrethroid insecticides tested. Whereas previous work demonstrated that the resistance to Cry1Ac in the strain analyzed here is conferred by a mutation disrupting an ATP-binding cassette protein named ABCC2, the new results show that increased susceptibility to abamectin is genetically linked with the same mutation. Moreover, RNAi silencing of *HaABCC2* not only decreased susceptibility to Cry1Ac, it also increased susceptibility to abamectin. The mutation disrupting ABCC2 reduced removal of abamectin in live larvae and in transfected Hi5 cells. The results imply that negative cross-resistance occurs because the wild type ABCC2 protein plays a key role in conferring susceptibility to Cry1Ac and in decreasing susceptibility to abamectin. The negative cross-resistance between a Bt toxin and other bacterial insecticides reported here may facilitate more sustainable pest control.

## Author Summary

The soil bacterium *Bacillus thuringiensis* (Bt) produces proteins that kill insect pests but do not harm most other organisms including people. Extensive use of Bt proteins in sprays and genetically engineered crops selects for rapid evolution of resistance in pests, reducing economic and environmental advantages of this alternative to conventional insecticides. We discovered that resistance to Bt toxin Cry1Ac in the invasive crop pest *Helicoverpa armigera* increased susceptibility to abamectin and spineotram, insecticides derived from two other soil bacteria. Both resistance to Cry1Ac and increased susceptibility to abamectin are linked with the same mutation in a gene encoding a transporter protein ABCC2. The results imply that negative cross-resistance occurs because the wild type ABCC2 protein plays a key role in conferring susceptibility to Cry1Ac and in decreasing susceptibility to abamectin. The negative cross-resistance between a Bt toxin and other bacterial insecticides reported here may facilitate more sustainable pest control.

## Introduction

Insecticidal proteins from the bacterium *Bacillus thuringiensis* (Bt) are used widely in sprays and transgenic plants to control insects that attack crops and vector diseases [1,2]. These Bt proteins are especially valuable because they kill some devastating pests, but are not toxic to humans and most other organisms [1,3–6]. Farmers planted corn, cotton and soybean genetically engineered to produce Bt proteins on 78 million hectares worldwide in 2014, with a cumulative total of 648 million hectares of Bt crops planted since 1996 [2]. In the United States, transgenic Bt plants accounted for 80% of the corn and 84% of the cotton grown in 2014 [7]. Although Bt crops have provided substantial economic and environmental benefits [1,8–12], evolution of pest resistance to Bt proteins can diminish or even eliminate these advantages [13–17].

To delay pest adaptation, many farmers have switched from transgenic crops producing only one Bt toxin to newer ones producing two or more Bt toxins that kill the same pest [18]. This “pyramid strategy” aims to use toxins sufficiently different so that evolution of resistance to one toxin does not confer cross-resistance to the others [18,19]. Unfortunately, cross-resistance is common between Bt toxins, often strong between closely related toxins and weak, yet generally positive, between more distantly related toxins [16,18].

Because cross-resistance occurs between many Bt toxins, increasing the sustainability of Bt crops requires integration of more diverse pest management tactics that are not undermined by cross-resistance. The ideal scenario is negative cross-resistance, where selection for resistance to a Bt toxin increases susceptibility to alternative controls [20,21]. When negative cross-resistance occurs, the alternative control imposes a fitness cost associated with Bt resistance that selects against Bt resistance [22]. Despite recognition that negative cross-resistance could greatly enhance sustainability, identifying practical, alternative controls that show negative cross-resistance with Bt toxins has remained elusive [20,21].

Among the diverse mechanisms causing resistance to Bt toxins [23,24], mutations disrupting the ATP-binding cassette (ABC) transporter protein named ABCC2 are genetically linked with resistance in strains of at least seven species of Lepidoptera including *Helicoverpa armigera* [25–28]. This insect recently invaded the New World and is one of the world’s most damaging crop pests. Previous work revealed a mutation in the gene encoding ABCC2 tightly linked with >1000-fold resistance to Bt toxin Cry1Ac in a laboratory-selected strain (LF60) relative to its unselected parent strain (LF) [28]. These results support the conclusion that the wild type ABCC2 plays an essential role in the mode of action of Cry1Ac against lepidopteran larvae [29,30]. Because the mutation in LF60 is expected to cause the loss of 143 amino acids, it is also likely to disrupt the normal function of ABCC2

In addition to recent reports implicating ABCC2 in susceptibility to Bt toxins, extensive evidence shows that many members of the superfamily of ABC transporter proteins protect cells by excreting xenobiotics, including ABC transporters that confer resistance to drugs and chemotherapy agents in humans and resistance to insecticides in arthropods [30–32]. Because some ABC transporter proteins protect cells from insecticides, we hypothesized that the disruption of ABCC2 conferring resistance to Cry1Ac would also increase its susceptibility to other insecticides. To test this hypothesis, we evaluated responses of the LF60 and LF strains of *H*. *armigera* to three conventional insecticides and two bacterial insecticides, abamectin (an avermectin) and spineotram (a spinosyn). Abamectin and spineotram are widely used neurotoxic insecticides derived from the soil bacteria *Streptomyces avermitilis* and *Saccharopolyspora spinosa*, respectively [33–35]. Abamectin acts via various ligand-gated ion chloride channels, such as glutamate-gated chloride channels [33,35,36] and spineotram acts on a subgroup of nicotinic acetylcholine receptors [34].

We discovered that laboratory selection for resistance to Cry1Ac increased susceptibility of the LF60 strain by 9.0-fold to abamectin and 2.6-fold to spinetoram, but did not affect susceptibility to endosulfan (a cyclodiene), phoxim (an organophosphate), or cyhalothrin (a pyrethroid). Analyses of inheritance, transcription, and abamectin concentration in larvae and in transfected Hi5 cells support the hypothesis that ABCC2 mediates the observed negative cross-resistance between Cry1Ac and abamectin.

## Results

### Evaluation of Cross-Resistance between Bt Toxin Cry1Ac and Five Insecticides

Selection for resistance to Cry1Ac significantly increased susceptibility to abamectin and spineotram of the LF60 strain of *H*. *armigera* relative to its unselected, Cry1Ac-susceptible parent strain LF (Tables 1 and 2). Relative to LF, the concentration killing 50% of larvae (LC_50_) for LF60 was 9.0 times lower for abamectin (Table 1) and 2.6 times lower for spineotram (Table 2). Selection of LF60 with Cry1Ac did not affect susceptibility to endosulfan, phoxim, or cyhalothrin (Table 2). The LC_50_ of abamectin was not higher for LF (1.23) than for the independently derived susceptible strain 96S (1.31), confirming that LF was not resistant to abamectin.

**Table 1:** Responses to abamectin in the Cry1Ac-resistant strain (LF60) of *H*. *armigera*, its Cry1Ac-susceptible parent strain (LF), and their F1 progeny; and in an independently derived susceptible strain (96S).

Strain or cross	LC_50_ ^a^ (95% FL)^b^	Slope (SE)^c^	Resistance ratio^d^
LF	1.23 (0.96–1.5)	1.3 (0.1)	1.0
LF60	0.137 (0.067–0.21)	1.6 (0.3)	0.11^e^
LF♀× LF60♂	0.515 (0.40–0.64)	1.6 (0.2)	0.42^e^ ^f^
LF♂× LF60♀	0.532 (0.41–0.66)	1.6 (0.2)	0.43^e^ ^f^
96S	1.31 (1.1–1.6)	1.5 (0.1)	1.1

^a^ Units are μg abamectin per ml diet

^b^ 95% fiducial limits

^c^ Slope of the concentration-mortality line and its standard error

^d^ LC_50_ of strain or cross divided by LC_50_ of LF

^e^ LC_50_ significantly lower than LF by non-overlap of 95% fiducial limits

^f^ LC_50_ significantly higher than LF60 by non-overlap of 95% fiducial limits

See S1 and S2 Tables for additional details.

**Table 2:** Responses to spineotram, endosulfan, phoxim, and cyhalothrin in the Cry1Ac-resistant strain (LF60) of *H*. *armigera* and its Cry1Ac-susceptible parent strain (LF).

Strain	Insecticide	LC_50_ (95% fiducial limits) (μg insecticide per ml diet)	Slope (SE)^a^	Resistance ratio^b^
LF	Spinetoram	0.112 (0.070–0.16)	2.5 (0.3)	1.0
LF60	Spinetoram	0.0432 (0.025–0.057)	2.3 (0.4)	0.38^c^
LF	Endosulfan	9.83 (3.2–23)	1.8 (0.2)	1.0
LF60	Endosulfan	8.66 (4.0–16)	1.9 (0.2)	0.88
LF	Phoxim	11.6 (3.0–50)	2.3 (0.2)	1.0
LF60	Phoxim	9.71 (4.9–23)	3.9 (0.3)	0.84
LF	Cyhalothrin	7.22 (4.73–10)	2.2 (0.2)	1.0
LF60	Cyhalothrin	7.49 (5.3–10)	2.1 (0.2)	1.0

^a^ Slope of the concentration-mortality line and its standard error

^b^ LC_50_ of strain divided by LC_50_ of LF

^c^ LC_50_ significantly lower for LF60 than LF by non-overlap of 95% fiducial limits

See S1 and S2 Tables for additional details.

### Inheritance of Increased Susceptibility to Abamectin

We conducted reciprocal crosses between LF and LF60 and tested the F1 progeny to evaluate inheritance of increased susceptibility to abamectin. The responses were similar between the F1 progeny from the two reciprocal crosses (Table 1), indicating that inheritance of susceptibility to abamectin is autosomal (i.e., no sex linkage or maternal effects). Based on the LC_50_ values for the F1 relative to the parent strains (Table 1), we calculated the dominance parameter (*h*), which varies from 0 for completely recessive to 1 for completely dominant [37]. For the two reciprocal crosses, *h* was 0.34 and 0.36, indicating that increased susceptibility to abamectin was a partially recessive trait.

**Fig 1:**
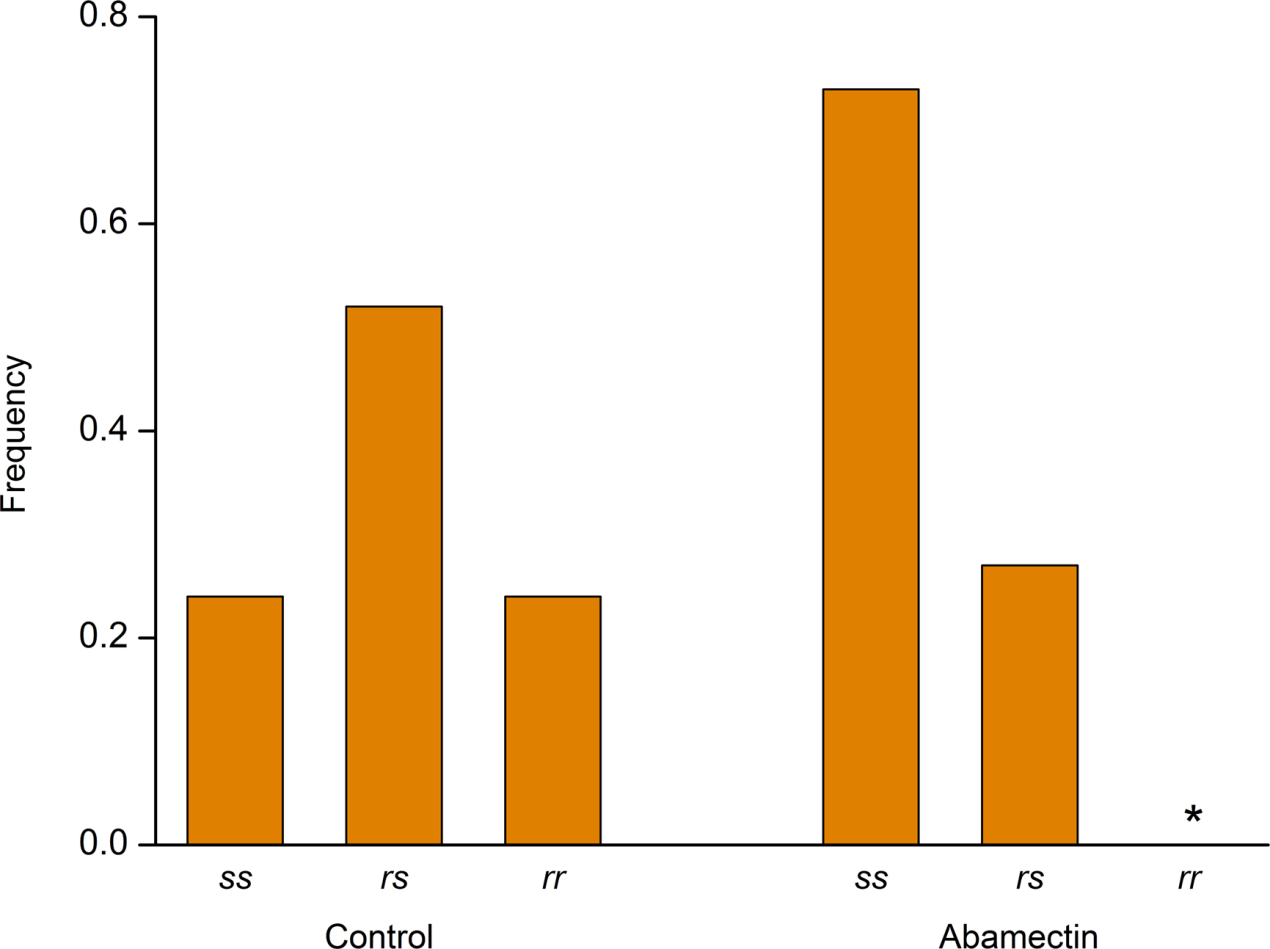
Genetic linkage between resistance to Cry1Ac and increased susceptibility to abamectin. Previous work showed that the *r* allele of the *HaABCC2* gene confers resistance to Cry1Ac and the *s* allele confers susceptibility [27]. The results here with F2 larvae from five single-pair families (total n = 200 larvae) show that on untreated diet (control), the genotype frequencies did not differ significantly from those expected under Mendelian inheritance (0.25 *ss*: 0.50 *rs*: 0.25 *rr)*, but on diet containing abamectin, the frequencies of *rr* and *rs* were significantly lower than expected (see text and S3 Table for details).

Analysis of F2 progeny shows that increased susceptibility to abamectin is genetically linked with the ABCC2 mutation that confers resistance to Cry1Ac (Fig 1 and S1 Table). We tested progeny from five single-pair F2 families that fed on either untreated diet or diet containing abamectin and sequenced genomic DNA individually for 20 survivors from each diet type. On untreated diet, the observed genotype frequencies at the *HaABCC2* locus were 0.24 *ss*: 0.52 *rs*: 0.24 *rr* (Fig 1 and S3 Table), which do not differ significantly from the frequencies expected under Mendelian inheritance (0.25 *ss*: 0.50 *rs*: 0.25 *rr*, Chi-squared = 0.08, df = 2, P = 0.96). However, on abamectin-treated diet, the genotype frequencies were 0.73 *ss*: 0.27 *rs*: 0.00 *rr*, which differ significantly from the expected frequencies (Chi-squared = 55, df = 2, P < 0.0001) (Fig 1 and S3 Table). Excluding the *rr*, which had 0% survival on treated diet, the results also show that relative to *ss*, the *rs* genotype was significantly lower than expected on treated diet (Chi-squared = 39, df = 1, *P* < 0.0001). Overall, the low survival on treated diet of *rs* and *rr* relative to *ss* demonstrates a strong association between increased susceptibility to abamectin and the *r* allele of *HaABCC2* that confers resistance to Cry1Ac.

### Variation in Transcription of *HaABCC2* among Tissues and Developmental Stages

Reversion transcription-polymerase chain reaction (RT-PCR) analysis of the LF strain showed that transcription of *HaABCC2* in 5^th^ instar larvae was much higher in the foregut, midgut and hindgut than in Malphigian tubules or cuticle (S1 Fig). Transcription of *HaABCC2* did not differ significantly between 4^th^ and 5^th^ instars, and was significantly higher in 4^th^ instars than in 1^st^ to 3^rd^ instars, pupae, and moths (S1 Fig).

### Silencing of *HaABCC2* with RNAi Decreases Susceptibility to Cry1Ac and Increases Susceptibility to Abamectin

**Fig 2:**
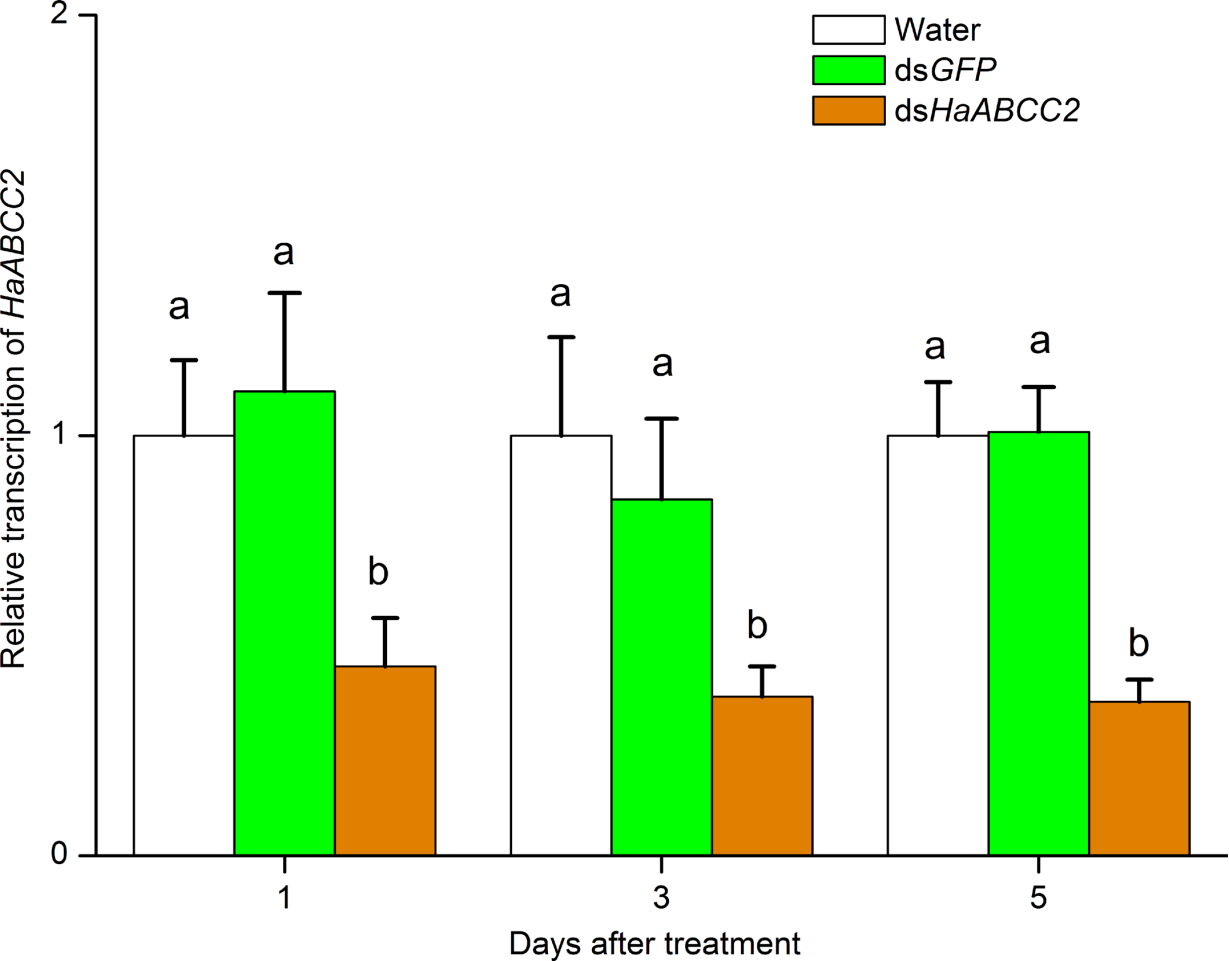
Suppression of *HaABCC2* transcription by RNAi in the Cry1Ac-susceptible LF strain of *H*. *armigera*. Early third instar larvae were fed individually with water (control), dsRNA from *GFP* (control) or dsRNA from *HaABCC2*. *HaABCC2* transcription was monitored using qRT-pCR at 1, 3 and 5 days after treatment. The bars show mean transcript levels relative to reference genes (actin and GAPDH) and standard errors from three biological replicates (n = 5 larvae per replicate). For 1, 3 or 5 days after treatment, different letters indicate significantly different means (P < 0.05 by Duncan’s multiple range tests).

**Fig 3:**
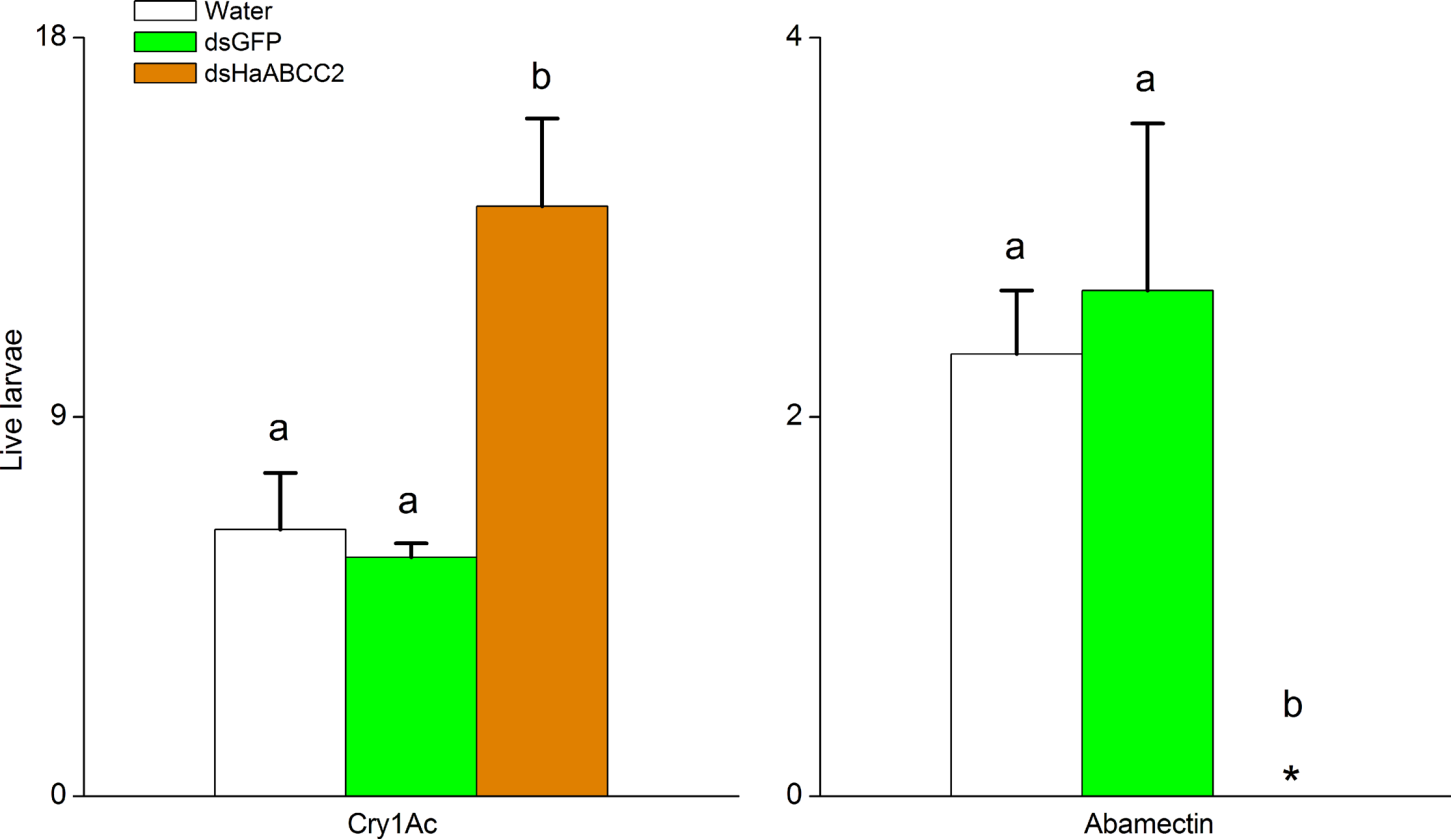
Silencing *HaABCC2* with RNAi decreased susceptibility to Cry1Ac and increased susceptibility to abamectin. After treatment with water, dsGFP, or dsHaABCC2, larvae from the Cry1Ac-susceptible LF strain of *H*. *armigera* were given diet containing Cry1Ac or abamectin (three replicates of 24 larvae for each treatment for each diet, total n = 432 larvae). Bars show means and their standard errors. The asterisk indicates no larvae treated with dsHaABCC2 survived on diet with abamectin. For each diet type (Cry1Ac or abamectin), different letters indicate significantly different means (P < 0.05 by Duncan’s multiple range tests).

For the Cry1Ac-susceptible strain LF, *HaABCC2* transcription was suppressed more than 50% for larvae fed droplets of water containing dsHaABCC2 relative to larvae fed control droplets with water only or dsGFP (Fig 2). However, this treatment of LF larvae with dsHaABCC2 did not affect transcription of two other genes, *HaABCC3* and *HaCAD* (S2 Fig). This suppression of *HaABCC2* transcription in LF larvae by RNAi significantly decreased susceptibility to Cry1Ac and increased susceptibility to abamectin (Fig 3 and S3 Fig). On untreated diet (control), treatment with dsHaABCC2 did not affect survival (S4 Fig).

### Higher Midgut Concentration of Abamectin Associated with Mutant *HaABCC2*

**Fig 4:**
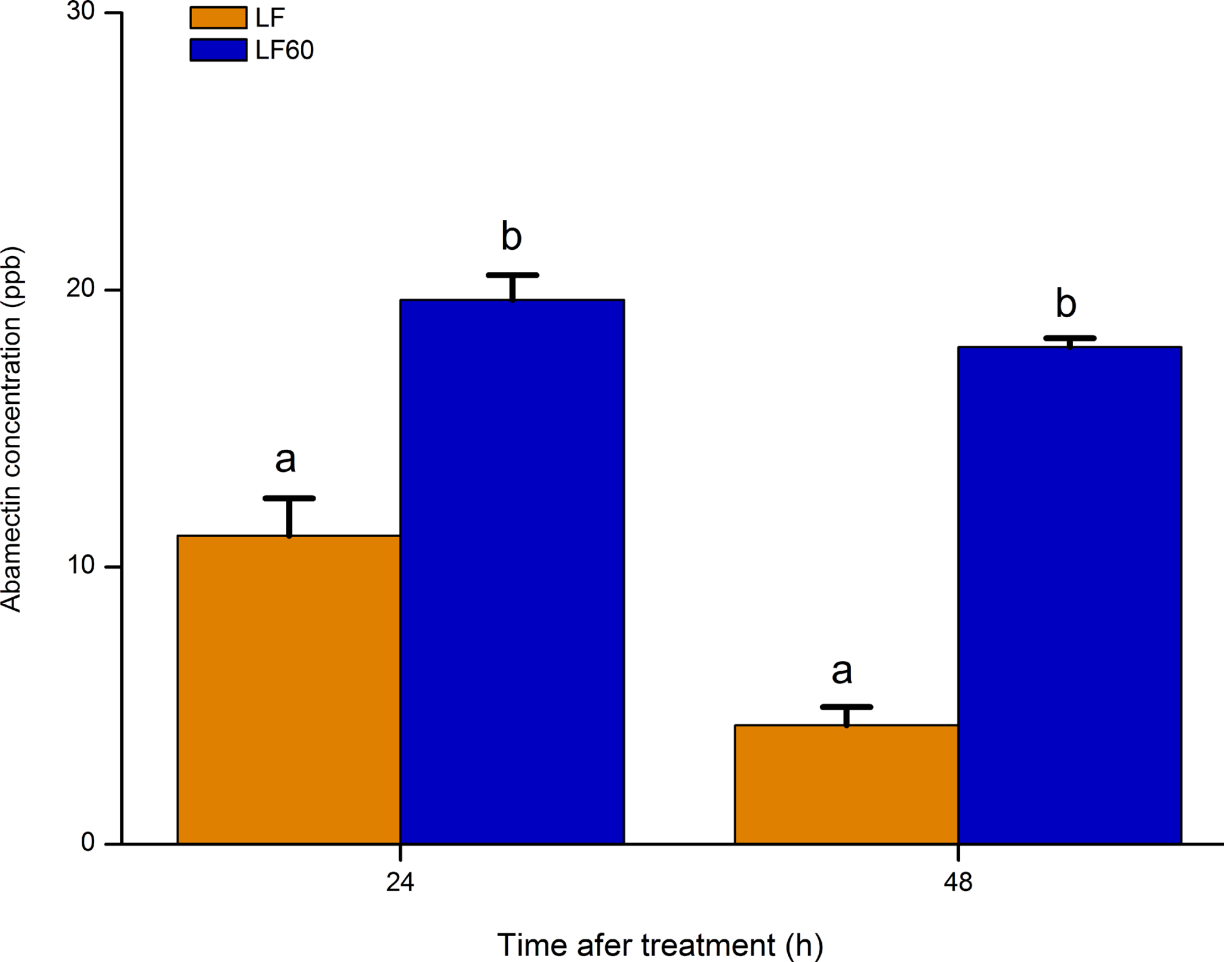
Concentration of abamectin in larval midgut for the Cry1Ac-resistant LF60 strain and the Cry1Ac-susceptible LF strain of *H*. *armigera*. Larvae from both strains fed on diet with 3 μg abamectin per ml. At 24 and 48 h after feeding on treated diet, the concentration of abamectin was significantly higher in LF60 than LF (t-tests, df = 4, 24 hours: t = 6.0, P = 0.004, 48 hours: t = 20.8, P < 0.0001).

We hypothesized that the mutant *HaABCC2* in LF60 increased susceptibility to abamectin by interfering with removal of abamectin. Consistent with this hypothesis, after larvae fed on diet containing abamectin, the concentration of abamectin in midgut tissues was significantly higher for LF60 (with mutant *HaABCC2*) than LF (with wild type *HaABCC2*), with a 2-fold difference after 24 h and a 4-fold difference after 48 h (Fig 4).

### Transfection of Cells with *HaABCC2* Increases Susceptibility to Cry1Ac and Decreases Abamectin Concentration

To test effects of *HaABCC2* directly, we transformed Hi5 cells with hybrid genes containing the *GFP* gene fused at the C-terminus of either the wild type *HaABCC2* gene from LF or the mutant *HaABCC2* from LF60 (*HaABCC2-GFP* and *mHaABCC2-GFP*, respectively). We also transfected Hi5 cells with only the *GFP* gene as a control. Western blots confirmed production of HaABCC2-GFP, mHaABCC2-GFP, and GFP respectively, in cells transfected with each of the three genes (S5 Fig).

**Fig 5:**
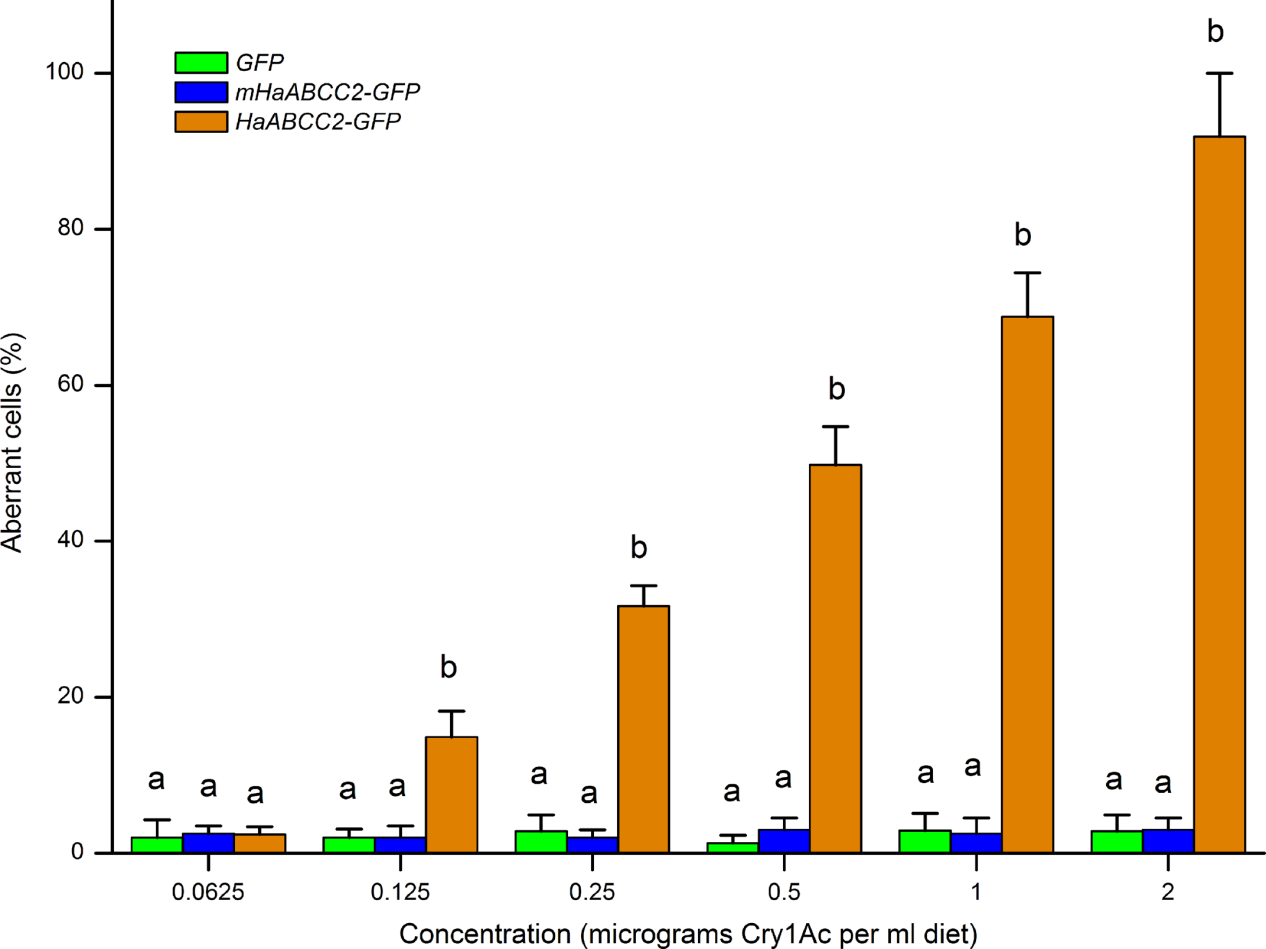
Transfection of Hi5 cells with the wild-type gene fused to GFP (*HaABCC2-GFP*), but not the mutant gene fused with GFP (*mHaABCC2-GFP*) or *GFP* alone, confers susceptibility to Cry1Ac. As the concentration increased from 0.0625 to 2 μg- Cry1Ac per ml, the percentage of aberrant cells increased from 2.4 to 91.9% for Hi5 cells transfected with *HaABCC2-GFP*, but remained ≤3% for cells transfected with *GFP* (control) or *mHaABCC2-GFP*. Different letters indicate significantly different means (P < 0.05 by Duncan’s multiple range tests).

**Fig 6:**
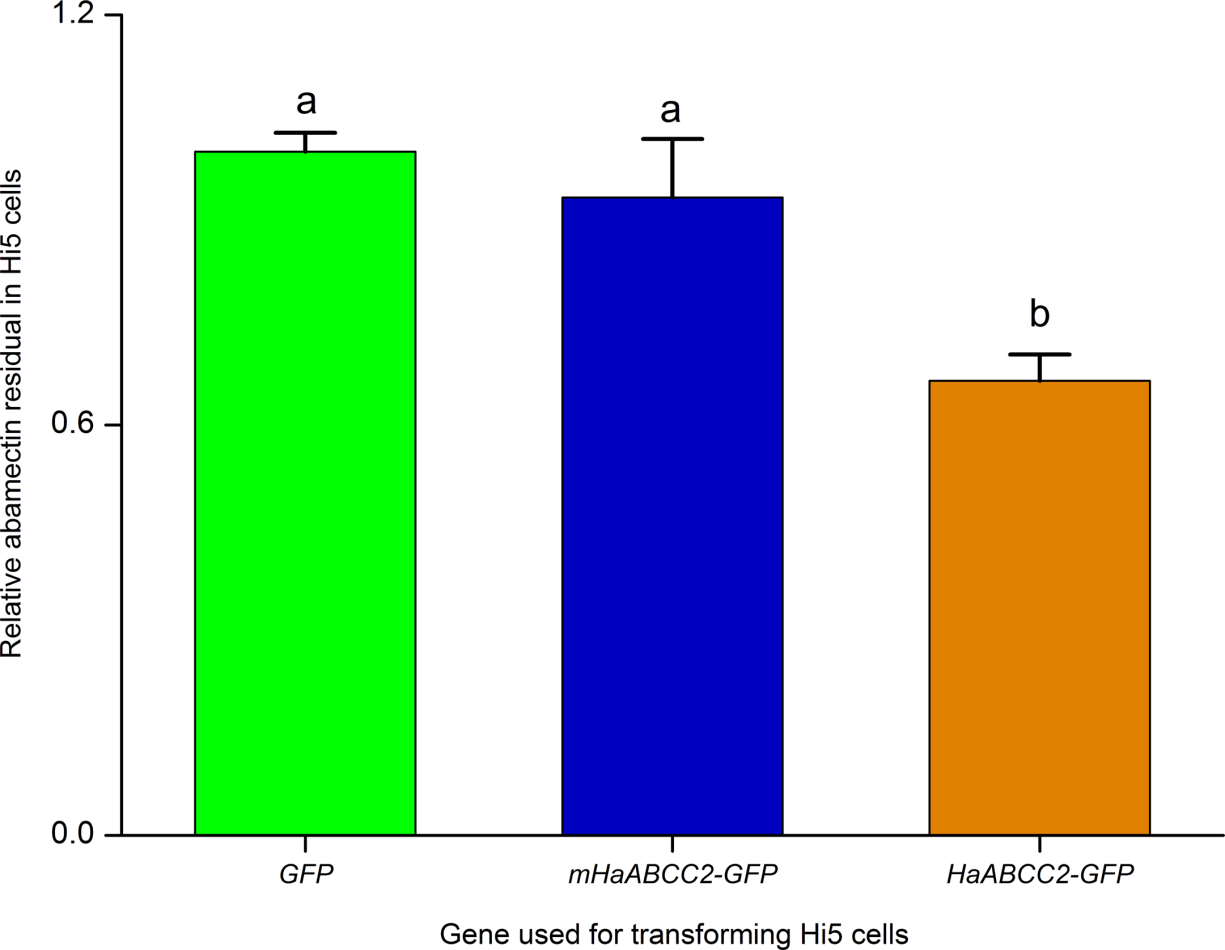
Transfection of Hi5 cells with wild type gene *HaABCC2-GFP* (brown) decreased residual abamectin concentration relative to Hi5 cells transfected with the mutant gene *mHaABCC2-GFP* (blue) or *GFP* (green). Different letters indicate significantly different means (P < 0.05 by Duncan’s multiple range tests).

Transfection of Hi5 cells with *HaABCC2-GFP*, but not with *mHaABCC2-GFP* or *GFP*, conferred susceptibility to Cry1Ac (Figs 5 and S6). We also found that after treating Hi5 cells with abamectin for 12 h, the concentration of abamectin was significantly lower in cells transfected with *HaABCC2-GFP* than in cells transfected with either *mHaABCC2-GFP* or *GFP* (Fig 6). These results are consistent with the results summarized above for the LF and LF60 strains implying that, relative to mutant *HaABCC2*, wild type *HaABCC2* increased susceptibility to Cry1Ac and the efflux of abamectin.

## Discussion

The results reported here show that selection for resistance to Bt toxin Cry1Ac in the LF60 strain of *H*. *armigera* increased its susceptibility to two other bacterial insecticides, abamectin and spineotram, but not to three conventional insecticides (endosulfan, a cyclodiene; phoxim, an organophosphate; and cyhalothrin; a pyrethroid) (Tables 1 and 2). Whereas previous work showed that the resistance to Cry1Ac in LF60 is tightly linked with a mutant allele that disrupts the ABC transporter protein ABCC2 [28], the new results here show that increased susceptibility to abamectin in LF60 is autosomal, partially recessive (mean *h =* 0.35), and linked with the same mutant allele (Fig 1 and S3 Table). Moreover, suppressing transcription of *HaABCC2* with RNAi both decreased susceptibility to Cry1Ac and increased susceptibility to abamectin (Fig 3). In addition, susceptibility of Hi5 cells to Cry1Ac was conferred by transfecting them with the wild type *HaABCC2* gene from the Cry1Ac-susceptible LF strain, but not with the mutant *HaABCC2* gene from LF60 (Figs 5 and S5). Finally, after exposure to abamectin, the concentration of abamectin was higher in midgut tissues of LF60 than LF, and higher in cells transfected with mutant *HaABCC2* from LF60 than in cells transformed with wild type *HaABCC2* (Figs 4 and 6). Collectively, these results imply that negative cross-resistance occurs in this case because the wild type ABCC2 protein in LF plays a key role in conferring susceptibility to Cry1Ac and decreasing susceptibility to abamectin by reducing the abamectin concentration in the midgut. Conversely, the evidence also suggests that the mutant ABCC2 protein in LF60 confers resistance to Cry1Ac by disrupting the mode of action of this Bt protein [28], while increasing susceptibility to abamectin by interfering with removal of abamectin by ABCC2.

An alternative hypothesis is that the decreased susceptibility to abamectin in LF relative to LF60 was caused by increased detoxification of abamectin in LF relative to LF60. We evaluated this alternative hypothesis because the results of Chen *et al*. [38] suggest that increased activity of some general detoxification enzymes accounted for a small portion of the 822-fold resistance to abamectin generated by their laboratory selection of *H*. *armigera* with abamectin. However, LF was not resistant to abamectin relative to the susceptible 96S strain (Table 1). Unlike the abamectin-selected strain analyzed by Chen *et al*. [38], LF had been reared in the laboratory without exposure to any insecticide for more than 15 years and was unlikely to have increased detoxification activity. In addition, relative to LF60, the LF strain did not have significantly decreased susceptibility to endosulfan, phoxim, or cyhalothrin (Table 2), which does not support the idea of increased general detoxification activity in LF relative to LF60. Most importantly, our analyses of genetic linkage, suppression of transcription by RNAi, and residual abamectin concentration in larvae and in transfected Hi5 cells provide strong evidence that ABCC2 mediates the observed negative cross-resistance between Cry1Ac and abamectin.

In related work from Australia, resistance to Bt toxin Cry2Ab in *H*. *armigera* was associated with a 1.5-fold increase in susceptibility to emamectin benzoate, an insecticide derived from abamectin, and a five-fold increase in susceptibility to an organophosphate (chlorpyrifos) and a carbamate (methomyl) [39]. Similarly, resistance to Cry2Ab in *Helicoverpa punctigera* was associated with increases in susceptibility of 1.2-fold to abamectin, 1.8-fold to chlorpyrifos, and 3.9-fold to methomyl [39]. In both species, resistance to Cry2Ab is genetically linked with mutations in the ABC transporter protein ABCA2 [40]. These results suggest that, similar to disruption of ABCC2 conferring resistance to Cry1Ac and negative cross-resistance to abamectin, the disruption of ABCA2 conferring resistance to Cry2Ab may cause the observed weak negative cross-resistance to avermectins (abamectin and emamectin benzoate) and stronger negative cross-resistance to chlorpyrifos and methomyl.

In a related study from China, susceptibility to emamectin benzoate ranged from 4.5 to 11-fold higher in 16 field populations sampled in 2011 relative to the Cry1Ac-susceptible laboratory strain named SCD [41]. Many of these field populations had been exposed to Bt cotton producing Cry1Ac, and several had minor, but statistically significant resistance to Cry1Ac relative to SCD [17,42].

Because disruption of ABCC2 is only one of the many mechanisms of resistance to Cry1Ac in *H*. *armigera* [17,24,28,43–45], the extent of negative cross-resistance between Cry1Ac and avermectins such as abamectin or emamectin benzoate may vary among populations or even within a population over time. Nonetheless, avermectins are considered effective as complements or alternatives to Bt toxins for control of this global crop pest because of their unique mode of action [46] and the typical absence of positive cross-resistance to avermectins caused by resistance to Bt toxins, organophosphates, carbamates, or pyrethroids [39,41,47,].

More generally, it will be important to determine if the ABCC2-mediated resistance to Cry1Ac in other major pests [25,26,48,49] causes negative cross-resistance to abamectin or other insecticides. It will also be useful to find out if the disruption of ABC transporters that confers resistance to Bt toxins increases insect susceptibility to other xenobiotics, such as plant defensive compounds. Because ABC transporters play a vital role in protecting insects from xenobiotics, their evolution may be constrained to preserve this function. The negative cross-resistance seen here between Bt toxin Cry1Ac and two other insecticides derived from soil bacteria, abamectin and spineotram, raises the intriguing possibility that Bt bacteria have exploited this constraint by targeting ABC transporter proteins and thereby delaying evolution of resistance to Bt toxins in their hosts. This delay could occur because the increased susceptibility to poisons from other bacteria such as *Streptomyces avermitilis* and *Saccharopolyspora spinosa* might be a major fitness cost associated with resistance to Bt toxins [22]. In any case, the negative cross-resistance reported here may facilitate more sustainable control of *H*. *armigera* and other pests because selection for resistance to Bt crops could increase their susceptibility to some insecticides.

## Materials and Methods

### Insect Strains and Rearing

The LF strain was started with larvae collected from Bt cotton in Langfang, Hebei Province, China in 1998 and was reared in the laboratory on artificial diet without exposure to Bt toxins or insecticides [50]. LF60 was generated by selecting insects from the susceptible LF strain with MVPII (Dow AgroSciences), a commercial formulation of Cry1Ac protoxin incorporated in the diet [45]. Selection was conducted for more than a decade with progressively increasing concentrations: 1, 5, 10, 30 and 60 μg Cry1Ac protoxin per g diet [45]. As an internal control, we also tested the susceptible strain 96S, which was started in 1996 from adults collected from conventional cotton in Xinxiang, Henan Province, China and reared in the laboratory on artificial diet without exposure to Bt toxins or insecticides [28, 50]. Rearing and bioassays were conducted at 27 ± 2°C, a photoperiod of 14L:10D, and 75 ± 10% relative humidity.

### Insecticide Bioassays

We used diet incorporation bioassays to test 2^nd^ instars against five insecticides [45] (purity and source given in parentheses): abamectin, cyhalothrin, and endosulfan (95%, 96%, and 90%, respectively, the pesticide factory of Institute of Plant Protection, Chinese Academy of Agricultural Sciences, Beijing, China), spineotram and phoxim (6% and 90%, BaySystems, Leverkusen, Germany). Spineotram was dissolved in distilled water and the other four insecticides were dissolved first in dimethyl sulfoxide (DMSO), and then in distilled water. For controls, we incorporated distilled water into diet for spineotram, and DMSO dissolved in distilled water for the other insecticides.

Larvae from the LF and LF60 strains were tested against a series of concentrations of each of the five insecticides. In addition, the F1 progeny from a cross between LF and LF60 were tested against abamectin. Mortality was recorded after 3 days and analyzed using probit methods (for details see Statistical Analysis below).

### Evaluation of Inheritance of Increased Susceptibility to Abamectin

We obtained 40 males and 40 female virgin moths from the LF and LF60 strains respectively, and set up two reciprocal mass crosses: 40 ♂LF x 40 ♀LF60 and 40 ♂LF60 x 40 ♀LF in plastic crates (5 L). Adults were allowed to mate and deposit eggs onto oviposition gauze. Newly hatched F_1_ neonates were transferred to individual 24 well-plate for bioassay.

### Genetic Linkage between *HaABCC2* and Susceptibility to Abamectin

We tested progeny from five single-pair F2 families, each generated by crossing a female F1 moth and a male F1 moth in paper cup (350 mL). We reared F2 larvae on untreated diet until they were early 2^nd^ instars and split them into two groups. For the next 3 days, one group of 50 larvae was provided untreated diet and the other group of 200 larvae was provided diet treated with 3 μg abamectin per ml. For 20 survivors from each type of diet, we sequenced genomic DNA of each individual (primers GP-F: TACTCTGCGATACAATTTGGA and GP-R: AGTACCACCTTCAGCTACTTT) at the *HaABCC2* locus to distinguish between the previously identified *r* allele harboring a mutation that confers resistance to Cry1Ac and the *s* allele that confers susceptibility to Cry1Ac [28].

### Variation in Transcription of *HaABCC2* among Tissues and Developmental Stages

We compared transcription of *HaABCC2* among tissues and development stages in the susceptible LF strain. For 5^th^ instar larvae, we compared the following five types of tissues: foregut, midgut, hindgut, malphigian tubules, and cuticle. For each biological replicate of each tissue type, we pooled tissues dissected under a dissecting microscope from 20 larvae. We also compared whole insects at the following five developmental stages (with age and sample size for each biological replicate in parentheses): 1^st^ instar (1 day, n = 80), 2^nd^ instar (3 days, n = 40), 3^rd^ instar (6 days, n = 30), 4^th^ instar (9 days, n = 15), 5^th^ instar (12 day, n = 10), pupae (4 days, n = 10), and adults (5 days, virgins, n = 10). All dissected tissues from 5^th^ instars and whole insects representing different developmental stages were quickly frozen in liquid nitrogen and stored at -80°C for subsequent RNA extraction. We used three biological replicates for each tissue type and development stage.

Total RNA was extracted from the sample homogenates according to the standard TRIzol reagent protocols (Invitrogen). RNA purity and concentration was evaluated by 260/280 and 260/230 ratios measured in a NanoDrop 3300 (Thermo). Total RNA (4 μg) was used for reverse transcription using SuperScript III First-Strand Synthesis for RT-PCR (Invitrogen), according to the manufacturer’s instructions in a final volume of 20 μl. The cDNA was diluted in nuclease free water for immediate use in qPCR or stored at -20°C.

We used quantitative real time PCR (qRT-PCR) to evaluate relative transcription of ABCC2 [28]. The thermal program of qPCR was 40 cycles of 95°C for 15 sec and 60°C for 34 sec. Gene expression was normalized relative to the reference genes of β-actin (Accession no. EU527017.1) and glyceraldehyde-3-phosphate dehydrogenase (GAPDH) (Accession no. JF417983.1). We calculated the relative copy number of mRNA using the method of 2^-ΔΔCt^ [51]. Primers and probes are shown in S4 Table.

### Suppression of *HaABCC2* with RNA Interference

We used RNA interference (RNAi) technique to test the role of HaABCC2 in susceptibility to Cry1Ac and abamectin in larvae from the susceptible LF strain [24]. We fed early third instars with dsRNA twice in 48 hours (At 0 hours and 48 hours) and determined transcript levels using qRT-PCR as described above at 24, 72 and 120 h after dsRNA-feeding at the second time. Feeding with water and dsRNA of green fluorescent protein (dsGFP) gene were used as controls.

The dsRNA was prepared using PCR products as template by *in vitro* transcription for RNAi. The primers for *HaABCC2* gene were: ABCC2 RNAiF 5’-TAA TAC GAC TCA CTA TAG TGG GCG ACT TTG GTG ATT TG’-3, ABCC2 RNAiR 5’-TAA TAC GAC TCA CTA TAT TTG ATG CTG CCG CTT ATG T’-3, and the green fluorescent protein (GFP) gene were: GFP RNAiF 5’-TAA TAC GAC TCA CTA TAG TCA AAG ATG ACG GGA ACT AC’-3, GFP RNAiR 5’-TAA TAC GAC TCA CTA TAC AAA CTC AAG AAG GAC CAT G’-3. T7 primer sequence was placed in front of both forward and reverse primers. *In vitro* transcription to produce dsRNA of *HaABCC2* and *GFP* genes were performed with T7 RNA polymerase using the HiScribe RNAi T7 In Vitro Transcription Kit (New England Biolabs) as reported by the manufacturer.

The dsRNA was used to feed 3^rd^ instar larvae of *H*. *armigera* as follows: larvae were individually placed into each well of 24-well plates to avoid cannibalism and starved for 12 h. Seventy two larvae were then fed with a 2 μl DEPC water drop containing 2.5 μg dsRNA from either *HaABCC2* and *GFP* respectively. After 2 h, droplet-fed larvae were placed back individually into 24-well plates provided with artificial diet. Two days later the dsRNA oral delivery was done one more time with a water drop containing 5 μg dsRNA as reported above. After feeding the dsRNA-fed larvae were placed individually into a 24-well plate provided with artificial diet with either 0 or 60 μg Cry1Ac/ml diet. To evaluate the silencing method, we assessed transcription of *HaABCC2* after 1, 2, and 3 days of dsRNA feeding. One insect was collected per sample, and quantitative RT–PCR was performed as described above. Each experiment and treatment consisted of five biological replicates. For these bioassays the 24 larvae previously fed with dsRNA were transferred individually into each vial containing diet either untreated diet or diet treated with 60 μg Cry1Ac protoxin/ml diet or 3 μg abamectin/ml diet and maintained under the rearing conditions described above. Survival, pupation and eclosion were scored every day.

### Abamectin Concentration in Larvae from LF and LF60

We provided individual middle 4^th^ instar larvae from LF and LF60 with 0.125g portions of diet containing 3 μg abamectin/ml. After all of the treated diet was eaten 6 h later, each larva was transferred to untreated diet for 24 h to make the larvae ingest the abamectin. 50 larvae were dissected and midgut of each larvae was taken out and washed in normal saline. After drying with absorbent paper, midgut were put in glass homogenizer (type76, Haimen BoTai, China) for fully homogenization, one min per homogenate and then put on ice cooling for one min. Homogenization was repeated until the midguts were completely smooth. 0.5g homogenated midgut tissue were transferred to a centrifuge tube (10 ml). We added 1 ml ultra-pure water and 2 ml acetonitrile, vortexing for 2 min added 0.5g NaCl and 1g anhydrous MgSO_4_ to the centrifuge tube, vortexing for 2 min. High speed (3500 RCF) centrifugation at 4°C for 5 min. The upper layer of the prepared sample was filtered using a 0.22 μm nylon syringe filter and transferred to an autosampler vial for injection. Area of abamectin in each injection were collected and used to calculate the concentration of abamectin in midgut of different insect lines and times according to the method of Du *et al*. [52] under the direction of Dr. Du in his laboratory. The analytical standard abamectin (95%) were from the pesticide factory of Institute of Plant Protection, Chinese Academy of Agricultural Sciences, Beijing, China. Chromatography grade acetonitrile and methanol were purchased from Honeywell International Inc. (New Jersey, USA). Acetonitrile for pesticide residue analysis was of analytical grade and purchased from Beijing Chemical Reagent Company (Beijing, China). Analytical grade sodium chloride (NaCl) and anhydrous magnesium sulfate (anhydrous MgSO4) were purchased from Beijing Chemical Company (Beijing, China). Ultra-pure water was obtained from a Milli-Q system (Bedford, MA, USA) [52]. The data was repeated in triplicate. Abamectin with the concentration of 1μg/ml was used for standard sample.

### Insect Cell Line

*Trichoplusia ni* BTI-Tn-5B1-4 (Hi5) cells were grown in Grace’s insect cell culture medium (Life Technologies Co., Grand Island, NY, USA) with 10% fetal bovine serum (FBS) (Life Technologies Co., Australia), 100 unit/ml penicillin 100 μg/ml streptomycin (Life Technologies Co., Grand Island, NY,USA) at 28°C [53].

### Construction of Plasmids and Over-expression of Fusion Proteins

Both *HaABCC2* from the susceptible larvae and the resistant strains were amplified by PCR using the specific primers (HaABCC2F: 5’-CTC AAG CTT CGA ATT CGC CAC CAT GGA AAA CGG TAC TAG TCC-3’; HaABCC2R: 5’-CCG CGG TAC CGT CGA CTG ACC GCC TCC GCC ACC GCC GTG GTG GTG GTG GTG GTG CT- 3’) and the corresponding pGEM T vectors inserted with the two genes as template. The fragments digested with *EcoR* I and *Sal* I were then inserted into plasmid pie2-EGFP-N1 at the corresponding sites, to generate plasmids pHaABCC2-GFP and pmHaABCC2-GFP. Both plasmids contained the pie2 promotor and the gene encoding GFP; pHaABCC2-GFP had the gene from LF encoding wild type ABCC2 and pmHaABCC2-GFP had the gene from LF60 encoding the mutant ABCC2. Hi5 cells were seeded into well of 6-wells culture plates at the density of 1× 10^6^ cells/well and grown overnight in Grace´s insect cell culture medium. We transfected each plasmid into Hi5 cells at 2μg/well using cell-fectin reagent (Life Technologies). At 24 h after transfection, the cells expressing recombinant proteins were washed with phosphate buffered saline (PBS), fixed in 4% paraformaldehyde for 20 min and stained with Hoechst 33342 (1 μg/ml) for 20 min at room temperature. The images were taken with a Nikon fluorescence microscope (E400).

### Western Blot

The Hi5 cells were seeded into 6-wells culture plate and transfected with plasmids pHaABCC2-GFP, pmHaABCC2-GFP and pGFP as described above, respectively. Cells were harvested by centrifugation and subjected to protein extraction at 24 h post transfection. The proteins were separated on 8% SDS-PAGE gel. After electrophoresis, the proteins were transferred onto PVDF membrane (Millipore Corporation, Billerica, MA, USA). The membrane was blocked with 5% non-fat milk in TBS-T buffer for 2 h at room temperature, and then incubated with mouse anti-GFP antibody (Abcam, Cambridge, UK) 1: 3000 dilution in TBS for 2 h at room temperature. The membranes were then washed for three times with TBS-T and then incubated with fluorescent secondary antibody 1:5000 dilution (Earthox, San Francisco, CA, USA). Finally, the membranes were washed for three times with TBS-T and bands were visualized using the Odyssey system (LI-COR Bioscience, Lincoln, NE, USA).

### Cytotoxicity of Cry1Ac Mediated by HaABCC2-GFP and the Mutant HaABCC2-GFP

Hi5 cells cultured into 96-well plates were transfected with plasmids pGFP, pHaABCC2-GFP and pmHaABCC2-GFP, respectively, according to the method [53]. The cells were washed twice with PBS at 24 h post transfection, then treated with the activated Cry1Ac toxin at the indicated concentrations for 1 h. The activated toxin was obtained by digesting protoxin (The State Key Laboratory for Biology of Plant Disease and Insect Pests, Institute of Plant Protection, Chinese Academy of Agricultural Sciences) [54] with trypsin (Sigma) at 37°C for 2 h at 20:1 (protoxin: trypsin) mass ratio.

The treated cells were observed and photographed under an inverted fluorescence microscope. The percentage of aberrant (swollen) cells were calculated according to the method of Tanaka *et al*. [55].

### Comparison of Abamectin Amount among HaABCC2-GFP-Expressing, Mutant HaABCC2-GFP-Expressing and GFP-Expressing Hi5 Cells

Hi5 cells were seeded into T75-flask and grown over night. Then the cells were transfected with plasmids pGFP, pHaABCC2-GFP and pmHaABCC2-GFP, respectively, and grown over night. Abamectin dissolved in DMSO was added into the flask at the final concentration of 5 μg abamectin/ml and incubated with the cells for 12 h. Then the cells were washed three times with PBS and collected by centrifugation at 500 g and 10 min. 0.05g cells were thawed five times and then transferred to a centrifuge tube (2 ml), we added 0.1 ml ultra-pure water and 0.2 ml acetonitrile, vortexing for 2 min. added 0.05g NaCl and 0.1g anhydrous MgSO_4_ to the centrifuge tube, vortexing for 2 min. High speed (3500 RCF) centrifugation at 4°C for 5 min. The upper layer of the prepared sample was filtered using a 0.22 μm nylon syringe filter and transferred to an autosampler vial for injection. The residuals of abamectin were then detected with the method of residuals of abamectin of insect midgut as described above. Area of abamectin in each injection were collected and used to calculate the concentration of abamectin in insect cells. These data were repeated in triplicate.

### Statistical Analysis

We used the SPSS Statistics (version 20.0) software (SPSS Inc.) to estimate the concentrations of insecticide killing 50% of larvae (LC_50_) and its 95% fiducial limits, and the slope of the concentration-mortality line and its standard error (Tables 1 and 2) [48]. Two values of LC_50_ were considered significantly different if there was no overlap between their 95% fiducial limits, which is a standard, but conservative criterion [56]. Because this criterion is conservative and this approach depends on fit of the data to the Probit model, we also analyzed the bioassay data using Fisher’s exact test (http://graphpad.com/quickcalcs/contingency2/), which is not conservative and does not rely on the Probit model. For each pairwise comparison, the conclusion about statistical significance did not differ between these two statistical approaches (S2 Table). We calculated the dominance parameter *h* from LC_50_ values as described previously [32]. In the genetic linkage analysis, we used chi-squared tests (http://vassarstats.net/newcs.html) to determine if the observed genotype frequencies differed significantly from the expected genotype frequencies (0.25 *rr*, 0.50 *rs*, and 0.25 *ss*) on untreated diet and on diet treated with abamectin. We used one-way analysis of variance (ANOVA) followed by Duncan’s multiple range test Statistica 6 (Statistica, SAS Institute Inc., Cary, NC, USA) to determine if means differed significantly among treatments in several experiments: among water, dsGFP, and dsHaABCC2 treatments for relative transcription of *HaABCC2* (Fig 2) and survival on diet treated with Cry1Ac, treated with abamectin, or untreated diet (control) (Figs 3, S3 and S4); among genes used for transfecting Hi5 cells (wild type *HaABCC2*, mutant *HaABCC2*, and *GFP*) in their effects on survival and abamectin concentration (Figs 5 and 6), among tissue types and developmental stages for transcription of *HaABCC2* (S1 Fig).

## Supporting Information

S1 Table Insecticide bioassays: Number of larvae tested including controls (n); and degrees of freedom (df), chi-squared (χ^2^) values and probability (P) values for goodness of fit from Probit analysis. (DOCX)

S2 Table Agreement between tests on statistically significant differences based on Fisher’s exact test (data below) and no overlap of the 95% fiducial limits of the LC_50_ values (data from Tables 1 and 2). (DOCX)

S3 Table Genetic linkage between *HaABCC2* and susceptibility to abamectin. (DOCX)

S4 Table Primers and probes used for RT-PCR (DOCX)

S1 Fig Transcription of *HaABCC2* in the LF strain of *H*. *armigera* determined by RT-PCR. (A) Variation among tissues of fifth instar larvae: foregut (FG), midgut (MG), hindgut (HG), Malphigian tubules (MT), and cuticle (CU). (B) Variation among developmental stages: larval instars (1^st^ through 5^th^), pupa, and moth. Actin and GAPDH genes were used as the reference genes to calculate relative transcription. RT-PCR was used to detect the expression levels of different samples, the mean relative transcript levels and corresponding standard errors determined from three biological replicates, all the mean expression levels are normalized in each Fig. For each panel (A and B), different letters indicate significantly different means (P < 0.05 by Duncan’s multiple range tests).
(TIF)

S2 Fig Effect of *HaABCC2* dsRNA on *HaABCC3* and *HaCAD* transcription in the Cry1Ac-susceptible *H*. *armigera* LF strain. Early third instar larvae were fed individually with water (control), dsRNA from *GFP* (control) or dsRNA from *HaABCC2*. *HaABCC3* and *HaCAD* transcriptions were monitored using qRT-pCR at 1, 3 and 5 days after treatment. The bars show mean transcript levels relative to two reference genes (actin and GAPDH) and standard errors from three biological replicates (n = 5 larvae per replicate). For 1, 3 or 5 days after treatment, different letters indicate significantly different means (P < 0.05 by Duncan’s multiple range tests).
(TIF)

S3 Fig Silencing *HaABCC2* with RNAi decreased susceptibility to Cry1Ac in the Cry1Ac-susceptible LF strain of *H*. *armigera*. After one of three treatments (water, dsGFP, or dsHaABCC2), larvae were given diet treated with Cry1Ac (three replicates of 24 larvae each for each treatment, total n = 216 larvae). We recorded normal pupae after 19 days and normal moths after 34 days. Bars show means and their standard errors. The asterisks indicate none of the larvae treated with water or dsGFP became normal moths. For each life stage (pupae or moths), different letters indicate significantly different means (P < 0.05 by Duncan’s multiple range tests).
(TIF)

S4 Fig On untreated diet (control), silencing *HaABCC2* with RNAi had no effect on survival of the Cry1Ac-susceptible LF strain of *H*. *armigera*. After treatment with water, dsGFP, or dsHaABCC2), larvae were fed with untreated diet (three replicates of 24 larvae each for each treatment, total n = 216 larvae). Live larvae were recorded after 9 days, normal pupae after 19 days, and normal moths after 32 days. Bars show means and their standard errors. No significant differences occurred among treatments for live larvae, normal pupae or normal moths (Duncan’s multiple range test for each of the three metrics).
(TIF)

S5 Fig Western blot of transfected Hi5 cells. Fusion proteins detected using anti-GFP antibody as primary antibody. Lane 1, protein size markers; lane 2, cells transfected with pGFP (control); lane 3, cells transfected with pHaABCC2-GFP; lane 4, cells transfected with pmHaABCC2-GFP (see text for details). Lanes 1–3 are from a single gel and lane 4 is from a different gel.
(TIF)

S6 Fig Expression of HaABCC2 confers susceptibility of Hi5 cells to Cry1Ac. In transfected Hi5 cells, HaABCC2 (wild type) from LF and mHaABCC2 (mutant) from LF60 occurred in both the cytoplasm and cell surface (A-D), while GFP occurred in the cytoplasm and nucleus (E). Thirty μg Cry1Ac per ml was not toxic to Hi5 cells expressing either GFP or mHaABCC2 (E and F), but 0.25 μg Cry1Ac per ml was toxic to Hi5 cells expressing HaABCC2 (G), showing that numerous cells swelled. The green cells expressing HaABCC2-GFP swelled and lysed more than those expressing GFP or mHaABCC2-GFP (H versus E or F). The gray images were obtained under white light microscopy, showing all cells. The color images were photographed under fluorescence microscopy, showing the cells emitting green fluorescence. Bar = 20 μm. White arrows point to cell membrane.
(TIF)

S7 Fig Quantification of residual abamectin concentration by ultra-performance liquid chromatography coupled with tandem mass spectrometry (UPLC-MS/MS) [52]. Representative multiple-reaction monitoring (MRM) chromatograms are shown for: Larval midgut tissue from (A) LF and (B) LF60; and for Hi5 cells transfected to produce: (C) GFP, (D) mutant HaABCC2 from LF60, and (E) wild type HaABCC2 from LF. Abamectin detection was based on the retention time (about 3 min) and molecular weight of 895.8. We used Masslynx NT V.4.1 (Waters, USA) software to collect and analyze the data. Data were obtained in triplicate (see Methods for details).
(TIF)
