# Supplementary material for: Resistance to Bacillus thuringiensis Mediated by an ABC Transporter Mutation Increases Susceptibility to Toxins from Other Bacteria in an Invasive Insect
Source: PLoS Pathog. 2016 Feb 12;12(2):e1005450. doi: 10.1371/journal.ppat.1005450 (PMC4752494; doi:10.1371/journal.ppat.1005450)
Supplement: S1 Table — (DOCX) [file ppat.1005450.s001.docx]

**Table S1**

| Strain or cross | Insecticide | n | df | χ^2^ | P |
| --- | --- | --- | --- | --- | --- |
| LF | Abamectin | 504 | 4 | 2.61 | 0.63 |
| LF60 | Abamectin | 504 | 4 | 1.44 | 0.84 |
| LF♀× LF60♂ | Abamectin | 504 | 4 | 5.26 | 0.26 |
| LF♂× LF60♀ | Abamectin | 504 | 4 | 6.24 | 0.18 |
| 96S | Abamectin | 504 | 4 | 5.78 | 0.22 |
| LF | Spinetoram | 432 | 3 | 5.36 | 0.15 |
| LF60 | Spinetoram | 432 | 3 | 4.09 | 0.25 |
| LF | Endosulfan | 504 | 4 | 33.2 | <0.001 |
| LF60 | Endosulfan | 504 | 4 | 22.8 | <0.001 |
| LF | Phoxim | 504 | 4 | 56.6 | <0.001 |
| LF60 | Phoxim | 504 | 4 | 40.0 | <0.001 |
| LF | Cyhalothrin | 504 | 4 | 10.1 | 0.039 |
| LF60 | Cyhalothrin | 504 | 4 | 7.22 | 0.125 |
