# Supplementary material for: Resistance to Bacillus thuringiensis Mediated by an ABC Transporter Mutation Increases Susceptibility to Toxins from Other Bacteria in an Invasive Insect
Source: PLoS Pathog. 2016 Feb 12;12(2):e1005450. doi: 10.1371/journal.ppat.1005450 (PMC4752494; doi:10.1371/journal.ppat.1005450)
Supplement: S2 Table — (DOCX) [file ppat.1005450.s002.docx]

**Table S2**

| Insecticide | Strain or F1^a^ | Mort.  (%)^b^ | Strain  or F1^a^ | Mort.  (%)^b^ | P^c^ | LC_50_  difference^d^ |  |  |
| --- | --- | --- | --- | --- | --- | --- | --- | --- |
| Abamectin | LF | 53 | LF60 | 89 | <0.001 | Yes |  |  |
| Abamectin | F1a | 69 | F1b | 70 | 0.77 | No |  |  |
| Abamectin | F1a | 69 | LF | 53 | <0.001 | Yes |  |  |
| Abamectin | F1a | 69 | LF60 | 89 | <0.001 | Yes |  |  |
| Abamectin | F1b | 70 | LF | 53 | <0.001 | Yes |  |  |
| Abamectin | F1b | 70 | LF60 | 89 | <0.001 | Yes |  |  |
| Abamectin | 96S | 52 | LF | 53 | 0.84 | No |  |  |
| Spineotram | LF | 71 | LF60 | 89 | <0.001 | Yes |  |  |
| Endosulfan | LF | 57 | LF60 | 60 | 0.41 | No |  |  |
| Phoxim | LF | 53 | LF60 | 58 | 0.06 | No |  |  |
| Cyhalothrin | LF | 65 | LF60 | 64 | 0.83 | No |  |  |

^a^ F1 progeny from crosses: F1a = LF♀× LF60♂, F1b = LF♂× LF60♀

^b^ Mortality percentage pooled across all concentrations tested (excluding control); for each comparison, both strains (or F1 progeny from crosses) were tested at the same concentrations with the same sample size per concentration (72).

^c^ Probability from Fisher’s exact test for independence of mortality between insect types

^d^ Significant difference between the insect types compared (strains or F1 progeny from crosses) based on no overlap of the 95% fiducial limits of the LC_50_ values (as reported in Tables 1 and 2).
