# Supplementary material for: Resistance to Bacillus thuringiensis Mediated by an ABC Transporter Mutation Increases Susceptibility to Toxins from Other Bacteria in an Invasive Insect
Source: PLoS Pathog. 2016 Feb 12;12(2):e1005450. doi: 10.1371/journal.ppat.1005450 (PMC4752494; doi:10.1371/journal.ppat.1005450)
Supplement: S3 Table — (DOCX) [file ppat.1005450.s003.docx]

**Table S3**

|  | Control diet | | |  | Treated diet | | |
| --- | --- | --- | --- | --- | --- | --- | --- |
| F2 family | *rr* | *rs* | *ss* |  | *rr* | *rs* | *ss* |
| A | 6 | 11 | 3 |  | 0 | 8 | 12 |
| B | 6 | 10 | 4 |  | 0 | 7 | 13 |
| C | 4 | 9 | 7 |  | 0 | 1 | 19 |
| D | 5 | 10 | 5 |  | 0 | 5 | 15 |
| E | 3 | 12 | 5 |  | 0 | 6 | 14 |
| Total | 24 | 52 | 24 |  | 0 | 27 | 73 |

We generated each of the five F2 families (A-E) by crossing an F1 male with an F1 female. We sequenced genomic DNA to determine the *HaABCC2* genotype for 40 larvae from each family (20 fed control diet and 20 fed diet treated with abamectin) to distinguish between the previously identified *r* and *s* alleles that confer resistance or susceptibility to Cry1Ac, respectively.
