# Supplementary material for: Resistance to Bacillus thuringiensis Mediated by an ABC Transporter Mutation Increases Susceptibility to Toxins from Other Bacteria in an Invasive Insect
Source: PLoS Pathog. 2016 Feb 12;12(2):e1005450. doi: 10.1371/journal.ppat.1005450 (PMC4752494; doi:10.1371/journal.ppat.1005450)
Supplement: S4 Table — (DOCX) [file ppat.1005450.s004.docx]

**Table S4**

| Gene name | Accession no. | Primers and probe sequences |
| --- | --- | --- |
| HaABCC2  HaABCC3  HaCAD | KF479231  KF479232  AF519180.2 | F: 5’- GGACAGTGAAATGACTCAGCAAGA-3’  R: 5’- CACATCATGGAGACGAAGTTCAG-3’  P: 5’-TGGCCTGTATGCTCTCGCCATGC-3’  F: 5’- GCGCGCATGTTAGTGGAGTT -3’  R: 5’- CCTTGGAACAAGTTAGGTTCTGTGT -3’  P: 5’- AGCGCATTCTGCAGTACACGCGC -3’  F: 5’- GAGCCTGGTCCAAGGAGTA-3’  R: 5’- CTCGAACACGGGCTCTC -3’  P: 5’- ACCGTAGTGTTTGTTCC -3’ |
| HaActin | X97615.1 | F: 5’-CCCGACGGACAGGTCATC-3’  R: 5’-GCCTCAGGGCAACGGAAT-3’  P: 5’-CCATCGGTAACGAAC-3’ |
| HaGAPDH | JF417983.1 | F: 5’-ACCATTGCCACCCAGAAGAC-3’  R: 5’-GCCATCACGCCACAGTTTG-3’  P: 5’-TGGATGGACCCTCTG-3’ |
