# Supplementary figures and images for: Resistance to Bacillus thuringiensis Mediated by an ABC Transporter Mutation Increases Susceptibility to Toxins from Other Bacteria in an Invasive Insect
Source: PLoS Pathog. 2016 Feb 12;12(2):e1005450. doi: 10.1371/journal.ppat.1005450 (PMC4752494; doi:10.1371/journal.ppat.1005450)

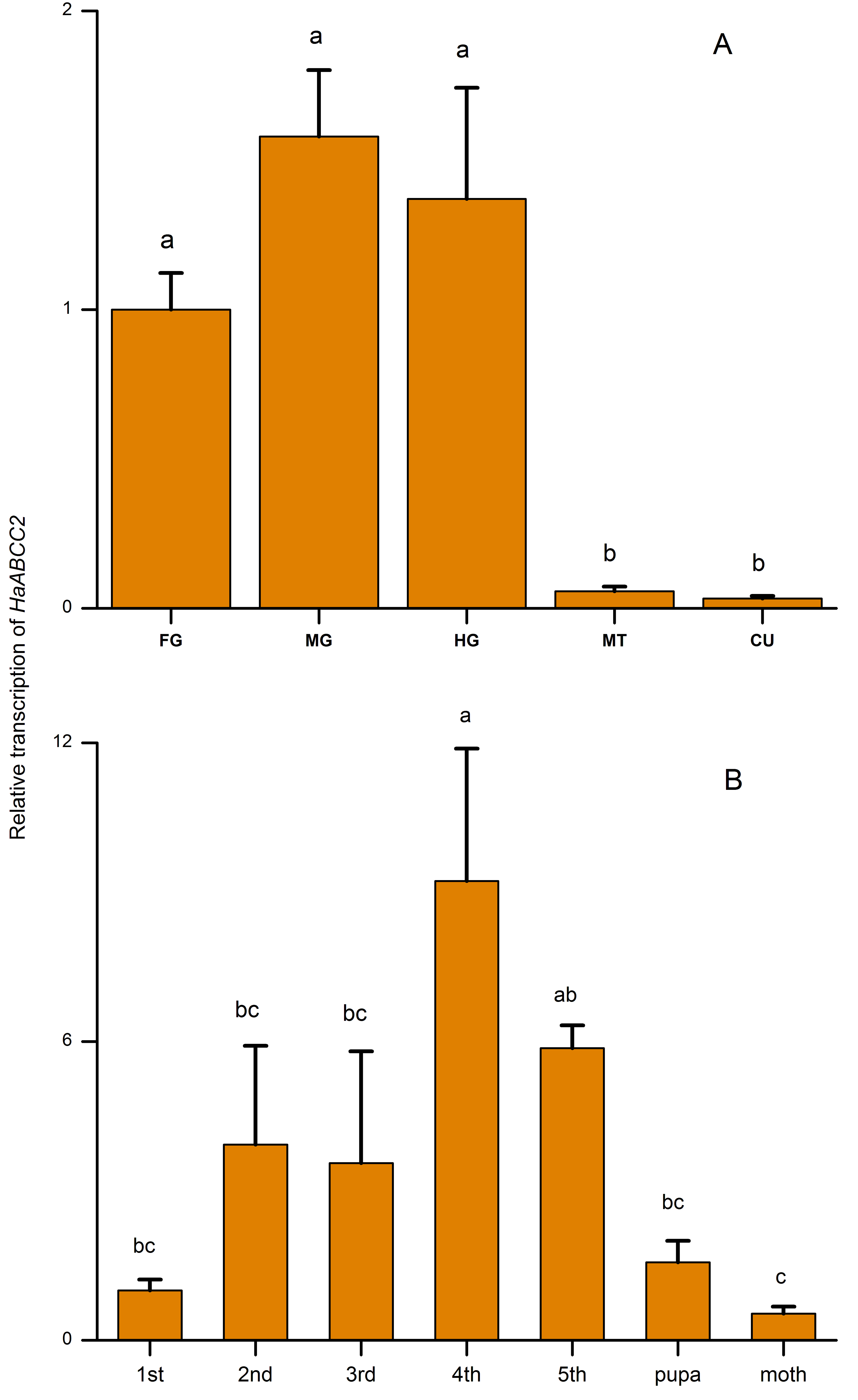

Supplement: S1 Fig — (A) Variation among tissues of fifth instar larvae: foregut (FG), midgut (MG), hindgut (HG), Malphigian tubules (MT), and cuticle (CU). (B) Variation among developmental stages: larval instars (1st through 5th), pupa, and moth. Actin and GAPDH genes were used as the reference genes to calculate relative transcription. RT-PCR was used to detect the expression levels of different samples, the mean relative transcript levels and corresponding standard errors determined from three biological replicates, all the mean expression levels are normalized in each Fig. For each panel (A and B), different letters indicate significantly different means (P < 0.05 by Duncan’s multiple range tests). (TIF) [file ppat.1005450.s005.tif]

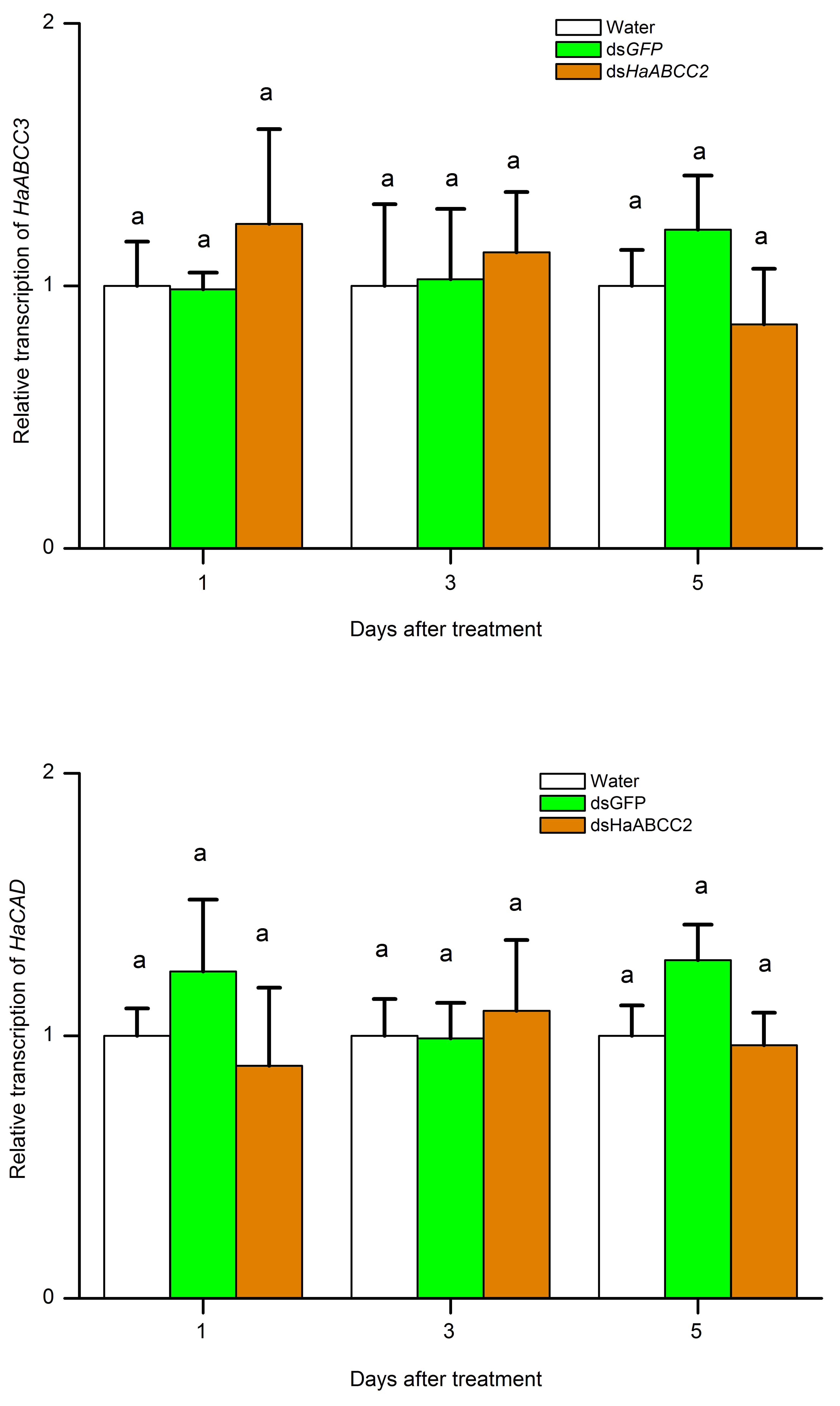

Supplement: S2 Fig — Early third instar larvae were fed individually with water (control), dsRNA from GFP (control) or dsRNA from HaABCC2. HaABCC3 and HaCAD transcriptions were monitored using qRT-pCR at 1, 3 and 5 days after treatment. The bars show mean transcript levels relative to two reference genes (actin and GAPDH) and standard errors from three biological replicates (n = 5 larvae per replicate). For 1, 3 or 5 days after treatment, different letters indicate significantly different means (P < 0.05 by Duncan’s multiple range tests). (TIF) [file ppat.1005450.s006.tif]

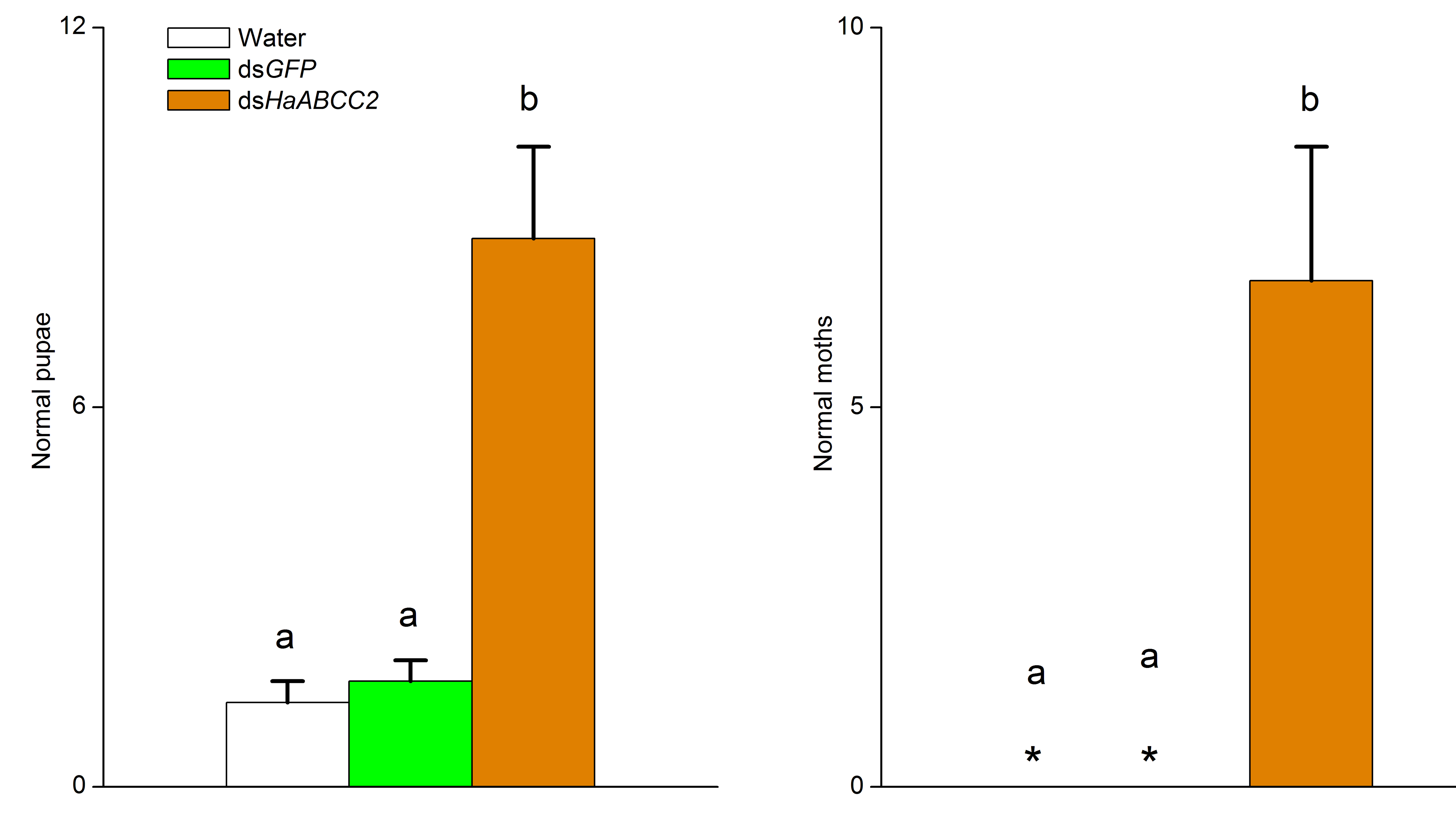

Supplement: S3 Fig — After one of three treatments (water, dsGFP, or dsHaABCC2), larvae were given diet treated with Cry1Ac (three replicates of 24 larvae each for each treatment, total n = 216 larvae). We recorded normal pupae after 19 days and normal moths after 34 days. Bars show means and their standard errors. The asterisks indicate none of the larvae treated with water or dsGFP became normal moths. For each life stage (pupae or moths), different letters indicate significantly different means (P < 0.05 by Duncan’s multiple range tests). (TIF) [file ppat.1005450.s007.tif]

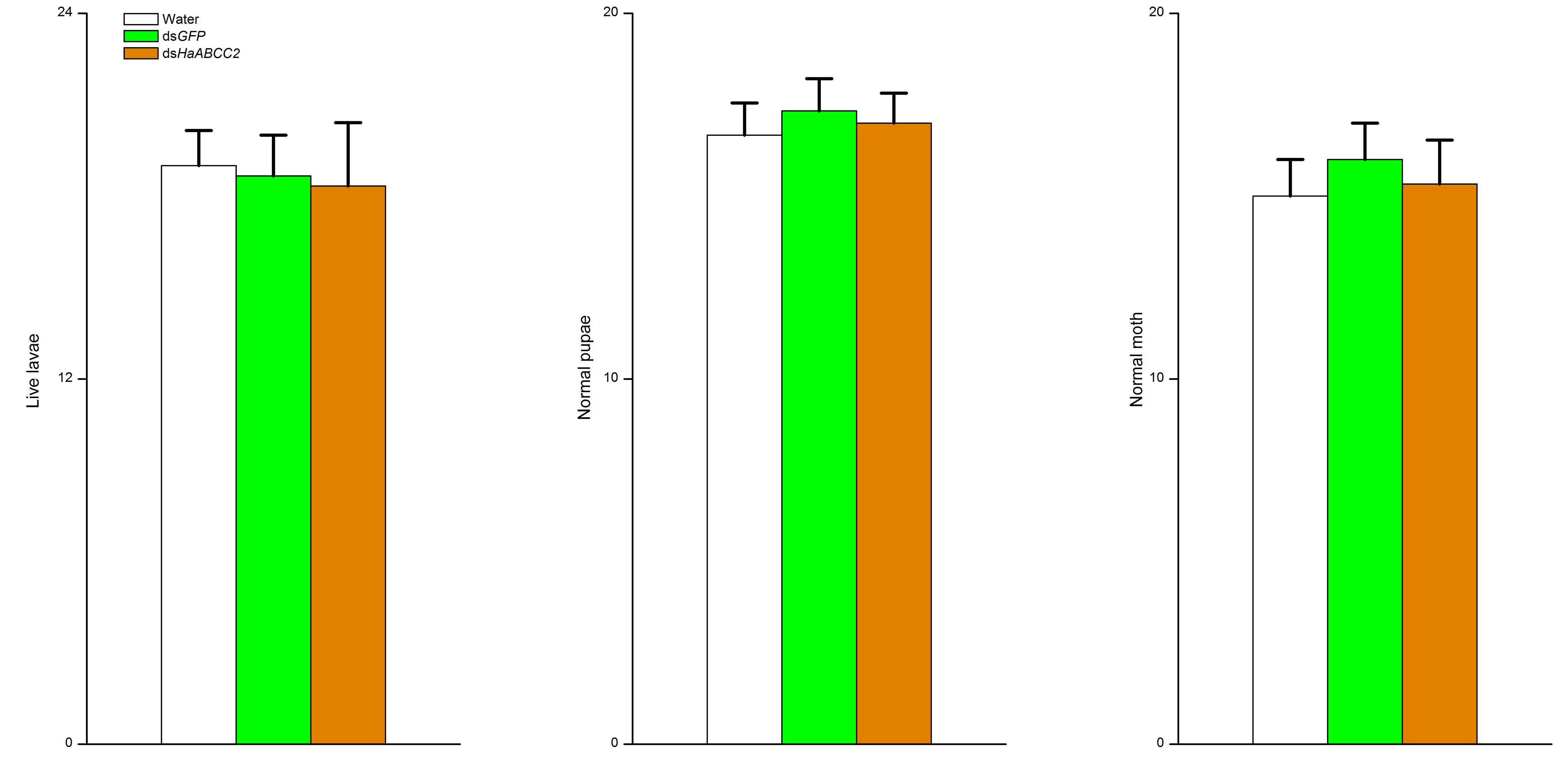

Supplement: S4 Fig — After treatment with water, dsGFP, or dsHaABCC2), larvae were fed with untreated diet (three replicates of 24 larvae each for each treatment, total n = 216 larvae). Live larvae were recorded after 9 days, normal pupae after 19 days, and normal moths after 32 days. Bars show means and their standard errors. No significant differences occurred among treatments for live larvae, normal pupae or normal moths (Duncan’s multiple range test for each of the three metrics). (TIF) [file ppat.1005450.s008.tif]

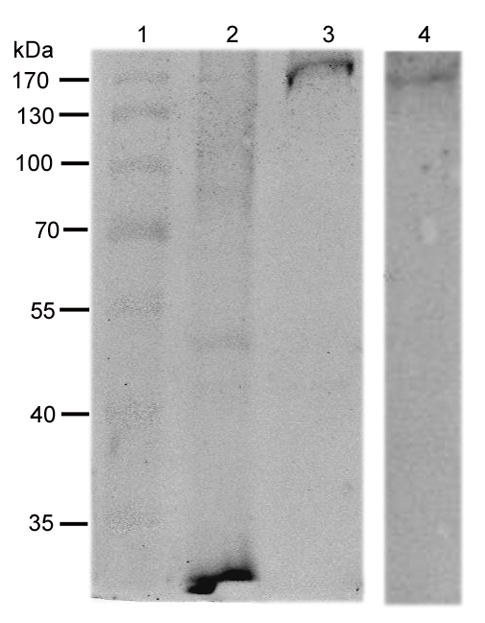

Supplement: S5 Fig — Fusion proteins detected using anti-GFP antibody as primary antibody. Lane 1, protein size markers; lane 2, cells transfected with pGFP (control); lane 3, cells transfected with pHaABCC2-GFP; lane 4, cells transfected with pmHaABCC2-GFP (see text for details). Lanes 1–3 are from a single gel and lane 4 is from a different gel. (TIF) [file ppat.1005450.s009.tif]

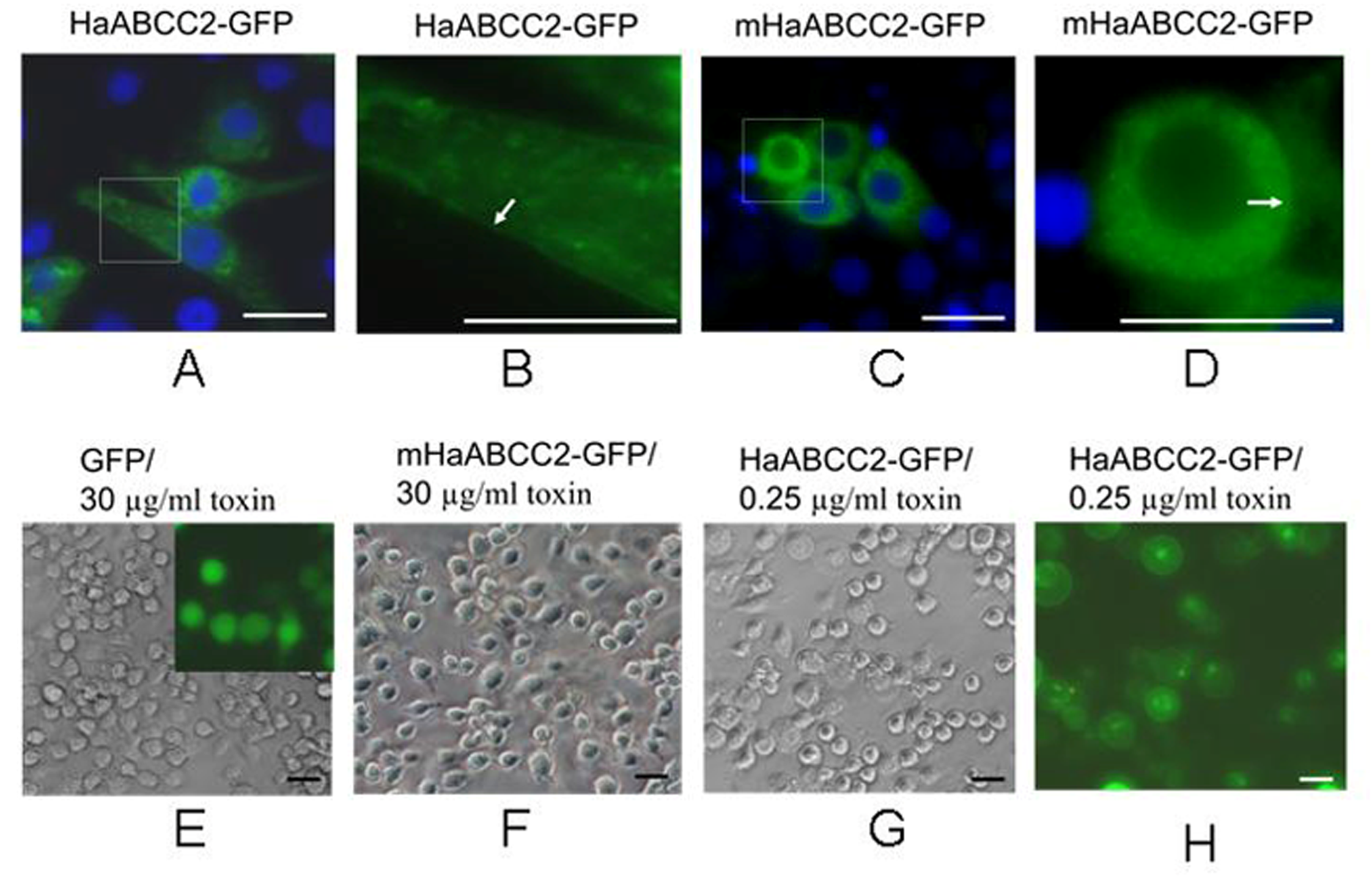

Supplement: S6 Fig — In transfected Hi5 cells, HaABCC2 (wild type) from LF and mHaABCC2 (mutant) from LF60 occurred in both the cytoplasm and cell surface (A-D), while GFP occurred in the cytoplasm and nucleus (E). Thirty μg Cry1Ac per ml was not toxic to Hi5 cells expressing either GFP or mHaABCC2 (E and F), but 0.25 μg Cry1Ac per ml was toxic to Hi5 cells expressing HaABCC2 (G), showing that numerous cells swelled. The green cells expressing HaABCC2-GFP swelled and lysed more than those expressing GFP or mHaABCC2-GFP (H versus E or F). The gray images were obtained under white light microscopy, showing all cells. The color images were photographed under fluorescence microscopy, showing the cells emitting green fluorescence. Bar = 20 μm. White arrows point to cell membrane. (TIF) [file ppat.1005450.s010.tif]

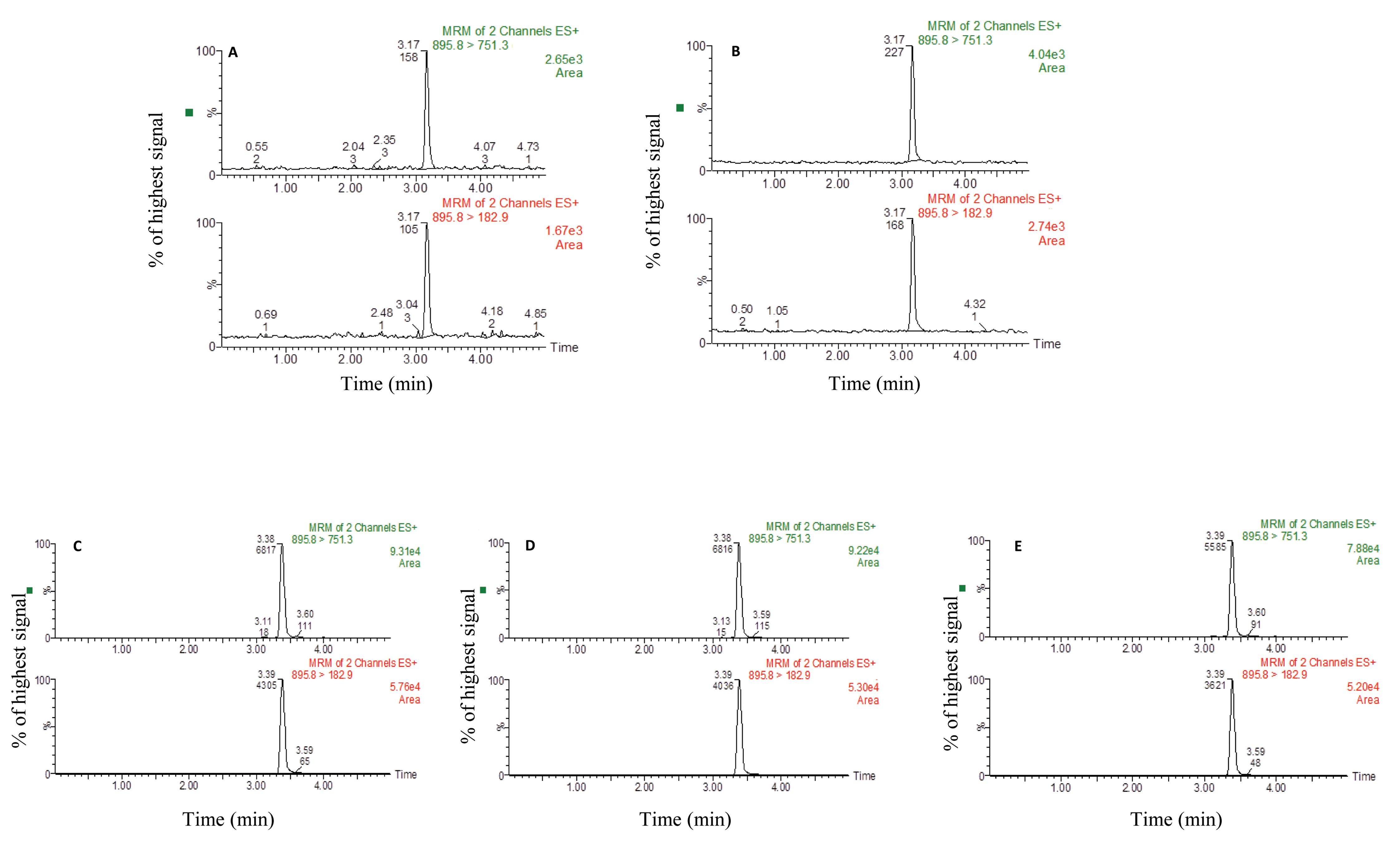

Supplement: S7 Fig — Representative multiple-reaction monitoring (MRM) chromatograms are shown for: Larval midgut tissue from (A) LF and (B) LF60; and for Hi5 cells transfected to produce: (C) GFP, (D) mutant HaABCC2 from LF60, and (E) wild type HaABCC2 from LF. Abamectin detection was based on the retention time (about 3 min) and molecular weight of 895.8. We used Masslynx NT V.4.1 (Waters, USA) software to collect and analyze the data. Data were obtained in triplicate (see Methods for details). (TIF) [file ppat.1005450.s011.tif]
